# Identification and Actions of a Novel Third Maresin Conjugate in Tissue Regeneration: MCTR3

**DOI:** 10.1371/journal.pone.0149319

**Published:** 2016-02-16

**Authors:** Jesmond Dalli, Julia M. Sanger, Ana R. Rodriguez, Nan Chiang, Bernd W. Spur, Charles N. Serhan

**Affiliations:** 1 Center for Experimental Therapeutics and Reperfusion Injury, Harvard Institutes of Medicine, Brigham and Women’s Hospital and Harvard Medical School, Boston, Massachusetts, 02115, United States of America; 2 Department of Cell Biology, Rowan University – SOM, Stratford, New Jersey, United States of America; University of Calgary, CANADA

## Abstract

Maresin conjugates in tissue regeneration (MCTR) are a new family of evolutionarily conserved chemical signals that orchestrate host responses to promote tissue regeneration and resolution of infections. Herein, we identified the novel MCTR3 and established rank order potencies and matched the stereochemistries of MCTR1, MCTR2 and MCTR3 using material prepared by total organic synthesis and mediators isolated from both mouse and human systems. MCTR3 was produced from endogenous substrate by *E*. *coli* activated human macrophages and identified in sepsis patients. Each of the three synthetic MCTR dose-dependently (1–100nM) accelerated tissue regeneration in planaria by 0.6–0.9 days. When administered at the onset or peak of inflammation, each of the MCTR promoted resolution of *E*. *coli* infections in mice. They increased bacterial phagocytosis by exudate leukocytes (~15–50%), limited neutrophil infiltration (~20–50%), promoted efferocytosis (~30%) and reduced eicosanoids. MCTR1 and MCTR2 upregulated human neutrophil and macrophage phagocytic responses where MCTR3 also proved to possess potent actions. These results establish the complete stereochemistry and rank order potencies for MCTR1, MCTR2 and MCTR3 that provide novel resolution moduli in regulating host responses to clear infections and promote tissue regeneration.

## Introduction

We recently identified, in self-resolving exudates, novel mediators that resolve infections [1–3] as well as regulate tissue regeneration [2]. These novel chemical signals link the resolution of infectious inflammatory exudates to the process of tissue regeneration. The acute inflammatory response, when self-contained, plays a critical role in limiting bacterial invasion to promote wound repair and tissue regeneration (for recent reviews, see [3, 4]). This protective response is orchestrated by autacoids at the site of inflammation [3, 5, 6]. Arachidonic acid-derived eicosanoids, namely prostaglandins and leukotrienes, are produced during the initiation stage of the inflammatory response leading to vascular leak and leukocyte recruitment [5, 6]. During resolution of acute inflammation, a process termed lipid mediator class switching occurs that leads to the formation of novel potent pro-resolving mediators [3]. This new genus of specialized pro-resolving mediators (SPM) include the n-3 essential fatty acid derived resolvins, protectins and maresins that possess leukocyte directed actions promoting the resolution of acute inflammation [1, 3, 7]. These mediators also stimulate the clearance of apoptotic cells and debris, playing fundamental role(s) in inflammation-resolution [3, 4, 8]. Resolution mediators such as Annexin A1 and Lipoxin A_4_ are critical in human control of tissue repair [9], and maresins in regeneration [2].

The biological actions of these pro-resolving mediators are stereochemically selective reflecting their routes of biosynthesis [3]. Therefore, establishing the complete stereochemical assignment and rank order potencies of lipid mediators are of considerable interest. Indeed establishing the complete stereochemistry of resolvins [3] enabled the confirmation and extension of the potent and diverse biological actions of these new mediators, for example, identification of Resolvin D1’s actions in treating postoperative cognitive decline [10] and protective actions of Maresin 1 (MaR1) in murine colitis [11].

Maresins (macrophage mediators in resolving inflammation) by definition are formed *via* 14-lipoxygenation of docosahexaenoic acid and promote resolution of acute inflammation and tissue regeneration [2, 3, 12]. Recently, we uncovered a new series of bioactive peptide-lipid conjugated mediators that are produced during the later stages of self-resolving infections [2] that are also present in human sepsis patients [13]. These were termed maresin conjugates in tissue regeneration (MCTR) because they regulate mechanisms in inflammation-resolution as well as tissue regeneration establishing links between local inflammatory exudates and tissue regeneration *via* novel chemical signals [2, 3]. The structure for MCTR1 is 13-glutathionyl, 14-hydroxy-docosahexaenoic acid, and that of MCTR2, 13-cysteinylglycinyl, 14-hydroxy-docosahexaenoic acid. In this report, we identified MCTR3 as 13-cysteinyl, 14-hydroxy-docosahexaenoic acid and established the potencies of MCTRs in the clearance of infections and tissue regeneration as well as their complete stereochemistry.

## Materials and Methods

### Lipid mediator metabololipidomics

All samples for LC-MS-MS-based lipidomics were subject to solid-phase extraction [13]. Prior to sample extraction, d_4_-LTB_4_, d_4_-PGE_2_ and d_5_-LTC_4_, representing each region in the chromatographic analysis (500 pg each), were added to permit quantification. Extracted samples were analyzed by a liquid chromatography-tandem mass spectrometry system, QTrap 5500 for eicosanoids (ABSciex) or QTrap 6500 (ABSciex) for MCTR equipped with an Agilent HP1100 binary pump and diode-array detector. An Agilent Eclipse Plus C18 column (100 mm × 4.6 mm × 1.8 μm) was used with a gradient of methanol/water/acetic acid of 80:20:0.01 (v/v/v) to 100:0:0.01 at 0.5 ml/min flow rate for eicosanoids and 55:45:0.1 (v/v/v) to 100:0:0.1 for MCTR. MCTR1, MCTR2 and MCTR3 were each prepared by total organic synthesis; purity of each was >98% and their structures confirmed using NMR [14].

To monitor and quantify the levels of targeted LMs, we used multiple reaction monitoring (MRM) with signature ion fragments for each molecule (six diagnostic ions and calibration curves). The following transitions were employed for MCTRs: MCTR1 m/z 650>191, MCTR2 521>191 and MCTR3 464>191 [13].

### Planaria tissue regeneration

Planaria (*Dugesia japonica*) were kept in water (Poland Spring; Nestle Waters North America, Stamford, CT, USA) at 18°C. All animals were starved for at least 7 days before the experiments. Tissue regeneration was assessed as described [2]. In brief, planaria were subjected to head resection postocularly (surgical injury). The posterior portions of the planaria were then placed in spring water containing 0.01% EtOH, or MCTR at indicated concentrations. The extent of tissue regeneration during a 6-day period was determined using captured images of regenerating blastemas at regular intervals (24 h). These images were analyzed using ImageJ software (NIH, Bethesda, MD, USA) to give a tissue regeneration index (TRI) [2].

### *E*. *coli* peritonitis

Peritonitis experiments were conducted as in [2]. Briefly, FVB mice (6-8-weeks old), purchased from Charles River Laboratories (Wilmington, MA, USA) were fed *ad libitum* Laboratory RodentDiet 20–5058 (LabDiet; Purina Mills, St. Louis, MO, USA) and housed at a maximum of four animals per cage and maintained in 12:12 hour light-dark cycles. Mouse experimental procedures were approved by the Standing Committee on Animals of Harvard Medical School (Protocol 02570) and complied with institutional and U.S. National Institutes of Health (NIH) guidelines. No surgical procedures were conducted and no analgesics were administered. Mice were euthanized by overdose of isoflurane via inhalation in saturated atmosphere, followed by cervical dislocation. If recovery from procedure is compromised as assessed by increase in self grooming, aggressive behavior or rapid movements, self-mutilation, absent activity, lack of eating or drinking, abdominal distention or redness and heat over abdominal area, humane endpoint in Protocol 02570 requires that mice must be euthanized immediately by isoflurane overdose, followed by cervical dislocation. During this procedure, animals did not experience more than slight or momentary pain or distress and no animals died prior to experimental endpoint. *E*. *coli* (serotypeO6:K2:H1) was cultured in Luria-Bertani broth and harvested at midlog phase (OD600 nm, ~0.5 absorbance units). In determined experiments mice were administered MCTR1, MCTR2, MCTR3 (50ng/mouse) or vehicle *via* intraperitoneal (*i*.*p*) injection and after 5 minutes inoculated with *E*. *coli* (10^5^ CFU/mouse). Exudates were collected at the indicated intervals and leukocyte populations as well as bacterial phagocytosis and macrophage efferocytosis determined using flow cytometry and fluorescent-labeled antibodies [2]. In select experiments, mice were inoculated with *E*. *coli* (10^5^ CFU/mouse), after 12h administered MCTR1, MCTR2, MCTR3 (100ng/mouse) or vehicle *i*.*p*. and exudates collected after 24h with leukocyte populations and bacterial phagocytosis determined as above.

### Phagocytosis

Human macrophages and neutrophils were prepared from peripheral blood leukocytes [2] and in accordance with the approved Partners Human Research Committee Protocol (1999P001297). Briefly, macrophages were plated in 96-well plates (5x10^4^ cells per well) for 24h, neutrophils were plated in 96-well plates (1x10^5^ cells per well) for 30 min and phagocytosis or efferocytosis was assessed as in [2]. For efferocytosis, apoptotic PMNs were obtained by culturing neutrophils overnight in PBS^-/-^ (5x10^6^ cells/ml). Apoptotic human PMNs were labeled with bisBenzimide trihydrochloride (Sigma-Aldrich). For efferocytosis, human macrophages were incubated with MCTR1, MCTR2, MCTR3 (1pM-10nM), or vehicle then labeled apoptotic PMNs (2.5x10^5^ cells) and incubated for 60 min (37°C pH 7.45). Fluorescence was measured using a SpectraMax M3 plate reader (Molecular Devices, Sunnyvale, CA, USA), and results were analyzed using SoftMax Pro (Molecular Devices). In select experiments, macrophages were incubated for 15 minutes with vehicle, MCTR1, MCTR2, MCTR3 (1pM-10nM) or a mixture of MCTR1+MCTR2, MCTR2+MCTR3, or MCTR1+MCTR3 (0.1nM each). BacLight Green Bacterial Stain (Life Technologies, Eugene, OR, USA) labeled *E*. *coli* (2.5x 10^6^ CFU/well) were added, cells incubated for 60 min (37°C pH 7.45) and fluorescence assessed using the fluorescence plate reader. In separate experiments, human neutrophils were incubated with MCTR1, MCTR2, MCTR3 (1pM-10nM), or vehicle then with BacLight Green Bacterial Stain-labeled *E*. *coli* (2.5x 10^6^ CFU/well; 60 min; 37°C pH 7.45) and fluorescence assessed as above.

Additionally, PMN survival was assessed. PMN were plated in 12-well plates at 5x10^6^ cells/mL in PBS with calcium and magnesium. At time zero, either PBS alone or 10nM MCTR1, MCTR2, or MCTR3 was added to PMN. Cells were incubated at 37°C with 5% CO_2_ for 24 or 48 hours, collected and analyzed by flow cytometry. Cells were surface-stained against Annexin V and Propidium Iodide (Molecular Probes, FITC AnnexinV/Dead Cell Apoptosis, Eugene, OR). Cell staining was evaluated using FACSCanto II flow cytometer (BD Biosciences, San Jose, CA) and results were analyzed using FlowJo software (Tree Star, Ashland, OR).

For real-time imaging, macrophages were plated onto 8-well chamber slides (0.1 x10^6^ cells/well in PBS^+/+^). The Nunc^™^ Lab-Tek^™^ II chamber system (Thermo Scientific, cat. # 15434) consists of a removable polystyrene media chamber attached to a glass microscope slide. The chamber slides were kept in a Stage Top Incubation system for microscopes equipped with a built-in digital gas mixer and temperature regulator (TOKAI HIT model INUF-K14). MCTR1, MCTR2 MCTR3 (1nM) or vehicle (PBS containing 0.01% EtOH) was added to macrophages for 15 min, followed by BacLight Green-labeled *E*. *coli* (2.5 x 10^6^ CFU). Images were then acquired every 6 min for 2h (37°C) with a Keyence BZ-9000 (BIOREVO) inverted fluorescence phase-contrast microscope (20X objective) equipped with a monochrome/color switching camera using BZ-II Viewer software (Keyence, Itasca, IL, USA). Green fluorescence intensity was quantified using BZ-II Analyzer.

### Statistical analysis

All results are expressed as the mean ± SEM. Differences between groups were compared using Student’s T-test (for two groups), 1-way ANOVA (multiple groups) followed by post hoc Bonferroni test, or 2-way ANOVA (multiple groups, multiple time points) followed by post hoc Bonferroni or Tukey tests as appropriate. The criterion for statistical significance was p < 0.05.

## Results

**Fig 1:**
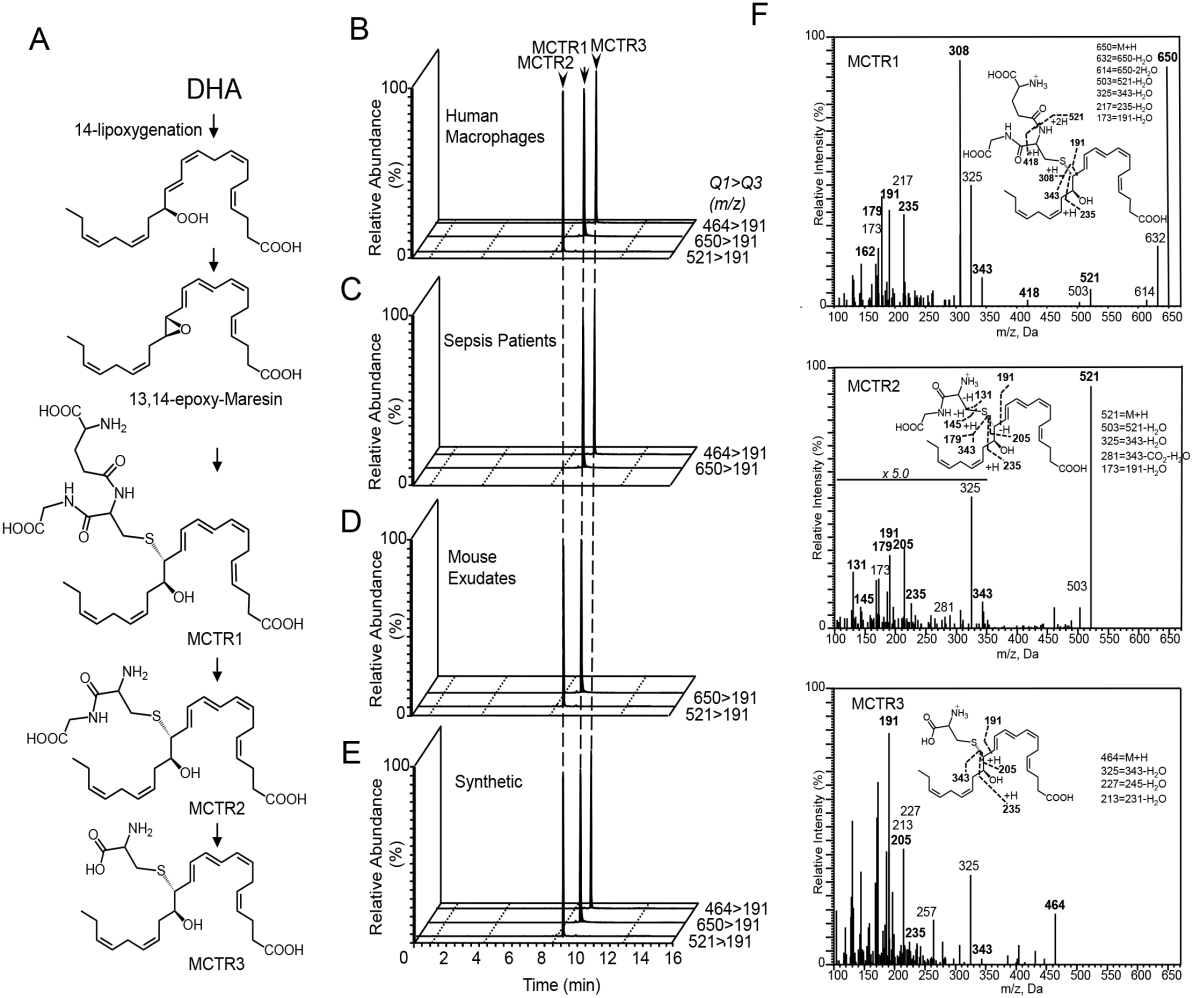
Endogenous MCTR from human macrophages, sepsis plasma and mouse infectious exudates match synthetic materials. (A) MCTR biosynthetic pathway. (B-D) Endogenous MCTR were obtained from (B) human macrophages (1x10^7^ cells/ml) incubated with *E*. *coli* (5x10^8^ CFU/ml; 30min, 37°C, PBS^+/+^; n = 3 macrophage preparations). (C) Sepsis plasma (n = 3 patients) (D) mice were inoculated with 10^5^ CFU/mouse *E*. *coli* and exudates collected after 24h (n = 3 mice). Products were extracted using C18 SPE and profiled using lipid mediator metabololipidomics. (E) Synthetic material. Results representative of 3 separate determinations. (F) MS-MS spectra for synthetic MCTR1, MCTR2 and MCTR3. Results are representative of d = 3.

**Fig 2:**
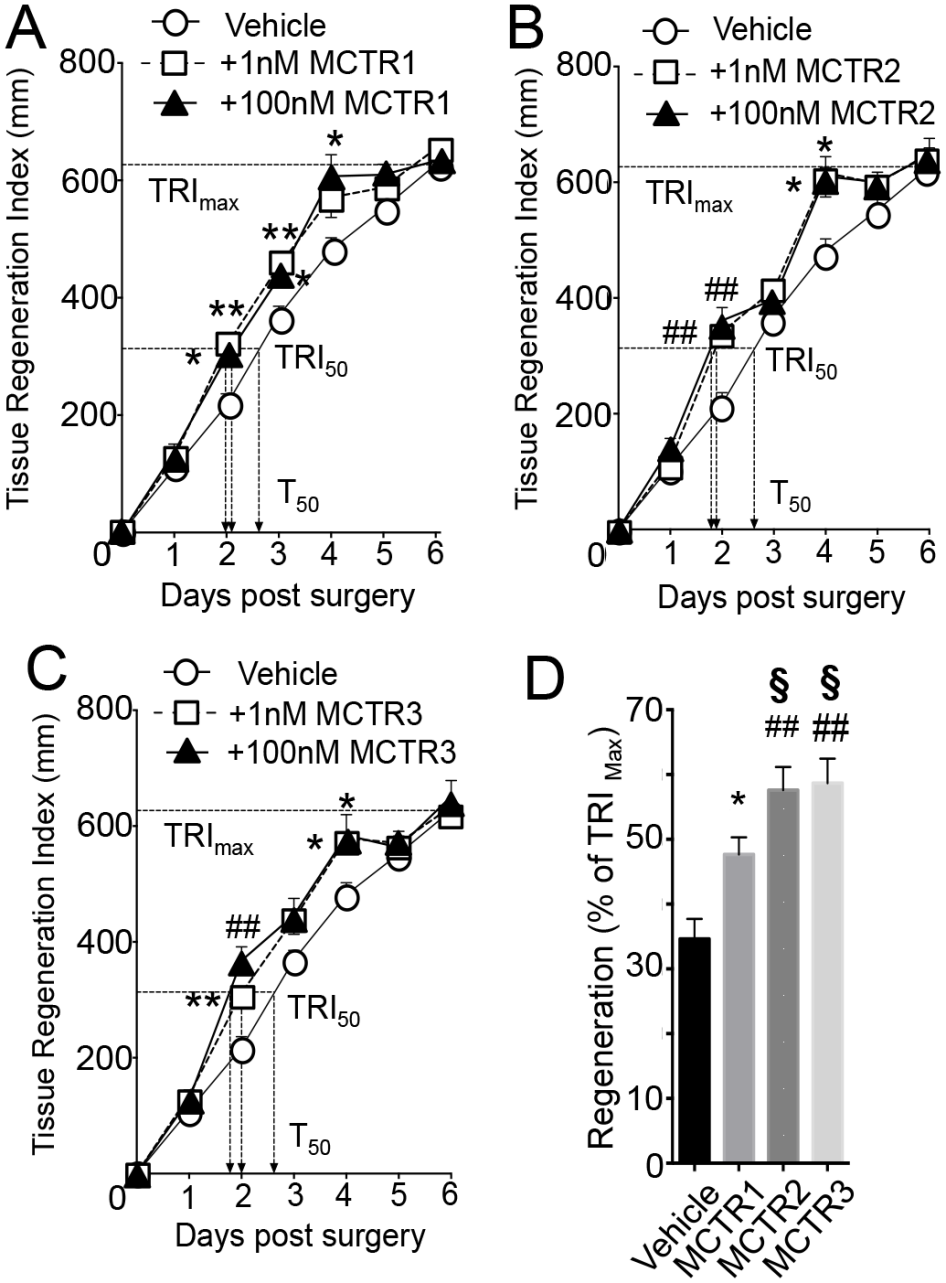
Synthetic MCTR dose dependently accelerate tissue regeneration in planaria. (A-C) Planaria were surgically injured, then kept in water containing (A) MCTR1 (1 or 100nM) (B) MCTR2 (1 or 100nM) (C) MCTR3 (1 or 100nM) or vehicle (water containing 0.01% EtOH), and tissue regeneration was assessed (see Materials and Methods for details). TRI_50_, time to 50% regeneration. (D) Rank order potency at promoting tissue regeneration measured at Day 2 post-surgery for 100nM MCTR. Results are mean ± SEM. n = 8 planaria per group. *p<0.05, **p<0.01, #p<0.001, ##p<0.0001 vs. surgical Injury + vehicle; §p<0.05 vs. surgical Injury + MCTR1.

We recently determined the complete stereochemistry of a key intermediate in maresin biosynthetic pathway 13S,14S-epoxy maresin [12], the proposed intermediate and immediate precursor in MCTR production (Fig 1A and [2]). Given that MCTR are produced in several biological systems, we obtained MCTR isolated from human macrophages, murine infectious exudates, and sepsis patients (Fig 1B–1D) to assess if they coeluted in LC-MS-MS and give MS-MS spectra identical to compounds independently prepared by total organic synthesis (Fig 1E). To determine the rank order potencies and stereochemistry for each MCTR, it was essential to use stereochemically pure precursors [14] because the endogenous mediators MCTR are produced in pico- to nanogram quantities that are not suitable for NMR analysis to obtain stereochemical assignments. In liquid chromatography tandem mass spectrometry, MCTR1 gave a distinct sharp peak with retention time (T_R_) = 9.8 min, MCTR2 gave a sharp peak with T_R_ = 9.1 min and the new MCTR3 gave a sharp peak with T_R_ = 10.3 min.

Co-injection of the synthetic with authentic MCTR1 demonstrated co-elution at T_R_ = 9.8 min (n = 3 macrophage preparations). Similarly, co-injection of synthetic MCTR2 and MCTR3 with MCTR isolated from human macrophages demonstrate co-elution with the respective authentic products (n = 3 macrophage preparations). Assessment of the MS-MS spectra for the material obtained by total organic synthesis gave essentially identical fragmentation spectra to those obtained with authentic material (Fig 1F and cf. [2, 13]). These mass spectra gave fragmentations and ions that were consistent with the deduced structures, where MCTR1 gave a parent ion with m/z of 650, MCTR2 an m/z of 521 and MCTR3 that of m/z 464. MCTR3 gave diagnostic ions at m/z 191, 205, 235 and 343 (see Fig 1F *inset*) as well as 464 (M+H), 325 (343-H_2_O), 227 (245-H_2_O), 213 (231-H_2_O), The ultraviolet chromophores for each of the three molecules were characteristic of conjugated triene double bond systems with an allylic auxochrome where the **λ**_max_^MeOH^ for MCTR1, MCTR2 and MCTR3 was 281nm. Thus, the stereochemically defined MCTR1, 2 and 3 each matched the endogenous molecules, permitting the identification of MCTR3.

Having matched the physical properties of synthetic, stereochemically defined materials with those of endogenously produced MCTRs from both human and murine systems, we next required assessment of their potent biological actions. Given that MCTRs display tissue regenerative actions, we assessed the ability of each synthetic MCTR to promote tissue regeneration. Planaria were surgically injured by rapidly removing the head portion of the animals and tissue regeneration was followed over time [2]. Planaria undergo both physiological and restorative regeneration via evolutionarily conserved pathways, making this an ideal system to characterize novel chemical signals involved in tissue regeneration [15]. When planaria were kept with vehicle alone, maximum tissue regeneration (TRI _max_) occurred within ~6 days with time to 50% regeneration (T_50_) occurring by ~2.7 days. Incubation of planaria with MCTR1 led to acceleration in tissue regeneration with a reduction in T_50_ from ~2.7 days to ~2 days when planaria were incubated with 100nM of MCTR1 and to ~1.9 days when incubated with 1nM (Fig 2A). MCTR2 also accelerated tissue regeneration with planaria, where incubation of surgically injured planaria with MCTR2 reduced T_50_ to ~1.8 days (Fig 2B) and at 1nM reduced T_50_ to ~1.9 days. The new MCTR3 also demonstrated potent tissue regenerative actions that were dose dependent (Fig 2C). Assessment of their relative potencies at stimulating tissue regeneration demonstrated that MCTR3 and MCTR2 were the most potent of the three, followed by MCTR1. Hence, MCTR3 ≈ MCTR2>MCTR1 (Fig 2D).

**Fig 3:**
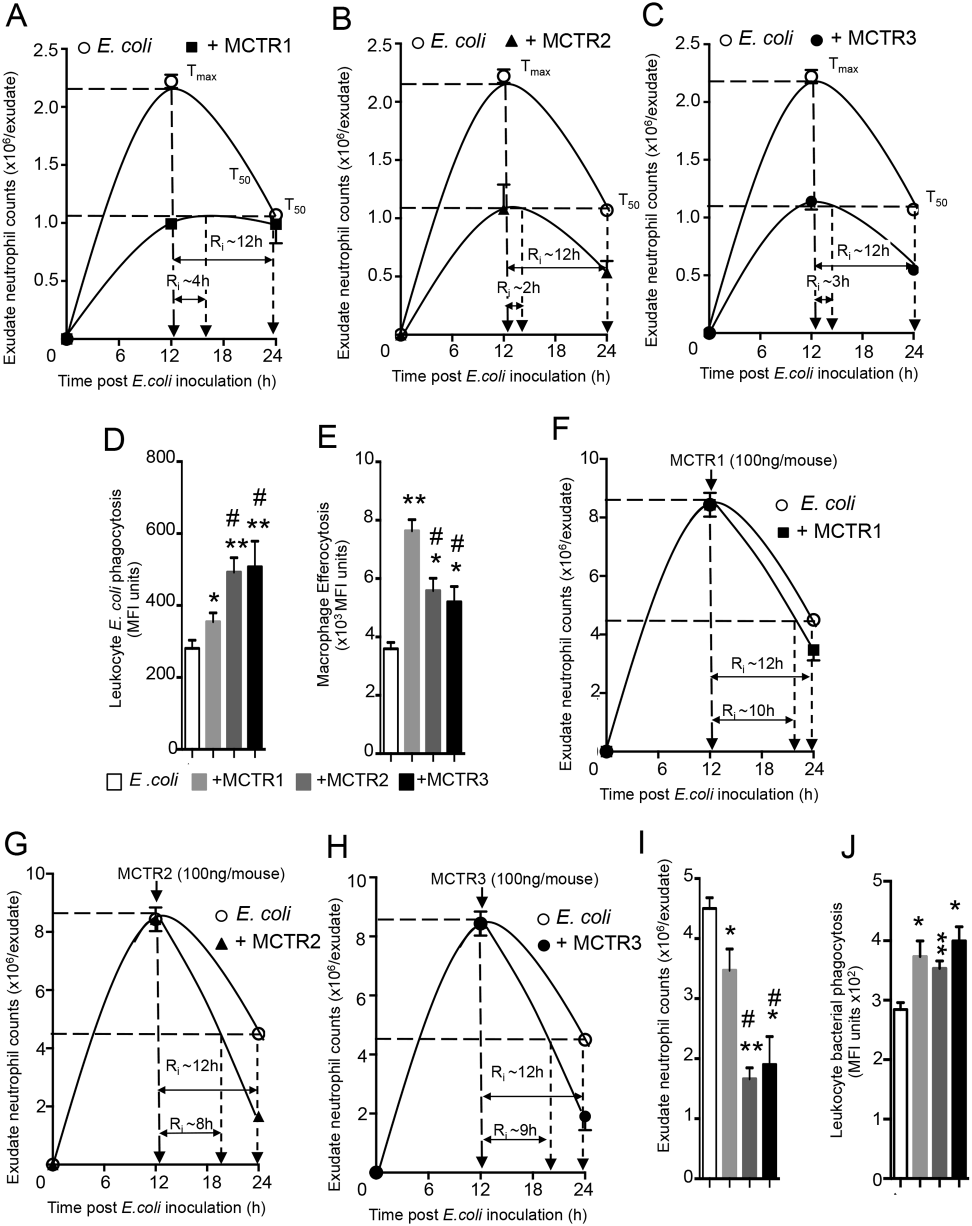
MCTRs accelerate resolution of *E*. *coli* infections. (A-E) Mice were administered (A) MCTR1, (B) MCTR2, (C) MCTR3 (50ng) or vehicle (saline containing 0.1% EtOH) via *i*.*p* injection; after 5 min they were inoculated with *E*. *coli* (10^5^ CFU/mouse) and exudates collected at the indicated time intervals; peritoneal leukocyte counts and resolution indices were determined (See Materials and Methods). (D) *E*. *coli* phagocytosis by exudate leukocytes was determined as median fluorescence units (MFI) in the CD11b^+^*E*. *coli*^+^ population at 12h (E) Exudate macrophage efferocytosis was determined as MFI in the F4/80^+^Ly6G^+^ population. (F-J) Mice were inoculated with *E*. *coli (*10^5^ CFU/mouse). After 12h, exudates were collected or (F) MCTR1, (G) MCTR2, (H) MCTR3 (100ng) or vehicle was administered *via i*.*p*. injection and peritoneal exudates collected 24h after *E*. *coli* inoculation; leukocyte counts were assessed and resolution indices determined. (I) 24h exudate leukocyte counts (see Materials and Methods). (J) *E*. *coli* phagocytosis by exudate leukocytes was determined as MFI in the CD11b^+^*E*. *coli*^+^ population. Results are mean±sem. n = 3 or 4 mice per group. *p<0.05, **p<0.01 vs. *E*. *coli* mice. #p<0.05 vs. *E*. *coli* + MCTR1.

Given the increased incidence of bacterial infections [1, 16] and that MCTR1 and MCTR2 each displays potent actions promoting resolution of infections, we could now test whether each of the stereochemically pure MCTR1, 2 and 3 also carried these actions in murine infections. *E*. *coli* (1x10^5^ CFU/mouse) inoculation gave a self-resolving inflammatory response with maximal neutrophil infiltration at ~12 h that subsequently declined, which gave a calculated resolution interval (R_i_) of ~12h (Fig 3A). MCTR1 (50ng/mouse) injection immediately prior to *E*. *coli* inoculation gave a significant reduction in neutrophil infiltration at the 12 h interval, and shortened the resolution interval from ~12h to ~4h (Fig 3A). Administration of MCTR2 also gave significant reductions in exudate leukocyte counts (~50%), a shortening in the R_i_ to ~2h and an increase in phagocytosis of *E*. *coli* (~50%) by exudate leukocytes. These results with synthetic MCTRs are in accordance with published findings with biologically produced MCTRs [2] and confirm the potent actions of these novel mediators in infections. Of note, MCTR3 given immediately prior to *E*. *coli* inoculation reduced exudate neutrophil levels by ~54%, shortening the R_i_ from 12 h to 3 h (Fig 3C). In addition to reducing neutrophil infiltration, each of three MCTR significantly increased leukocyte phagocytosis of *E*. *coli*, with MCTR2 and MCTR3 displaying the highest potencies (Fig 3D). MCTR1 was the most potent in stimulating macrophage efferocytosis (Fig 3E), a key defining pro-resolving action.

We also tested whether MCTR carry pro-resolving actions when administered at the peak of inflammation during *E*. *coli* infections. Administration of MCTR1 at the peak of inflammation (12h interval) gave reductions in exudate neutrophil counts at 24h and shortened R_i_ to ~10 h (Fig 3F). In mice given MCTR2 we found reductions in exudate neutrophil counts (~50%) giving an R_i_ of ~7 h (Fig 3G). At equal doses, MCTR3 also promoted the resolution of *E*. *coli* infections, reducing exudate neutrophil counts by ~50% and shortening the R_i_ to ~9 h (Fig 3G and 3H). Together these results demonstrate the potent biological actions of MCTR1 and MCTR2, confirming their original structural assignments (Fig 3I and 3J). Moreover, we describe here the unexpected actions of MCTR3 in accelerating the resolution of *E*. *coli* infections and establish the rank order potencies in promoting the resolution of infectious-inflammation.

During acute inflammation, classic eicosanoids can propagate the response by promoting vascular leakage (PGD_2_ and PGE_2_) and leukocyte recruitment (LTB_4_) and in some instances are associated with the switch to chronic inflammation [5, 6]. Given the potent bioactions of MCTR during infections, we assessed whether they regulated local eicosanoids during infections. MCTR1 at 100ng/mouse displayed the highest potency at counter-regulating exudate eicosanoids (n = 3 mice per group; p<0.05), significantly reduced PGD_2_ (~55%), PGE_2_ (~60%), PGF_2α_ (~50%), TxB_2_ (~50%) and LTB_4_ (~50%). MCTR2 gave a select regulation of eicosanoids including PGD_2_ (~70%) and PGF_2α_ (~35%) that were statistically reduced in the infectious exudates at the 12h interval, whereas MCTR3 significantly reduced PGD_2_ (~50%), PGE_2_ (~50%), PGF_2α_ (~50%) and TxB_2_ (~50%), thus suggesting that each of these novel molecules activates distinct counter-regulatory mechanisms in controlling exudate eicosanoids during infections (MCTR1>>MCTR3>MCTR2).

**Fig 4:**
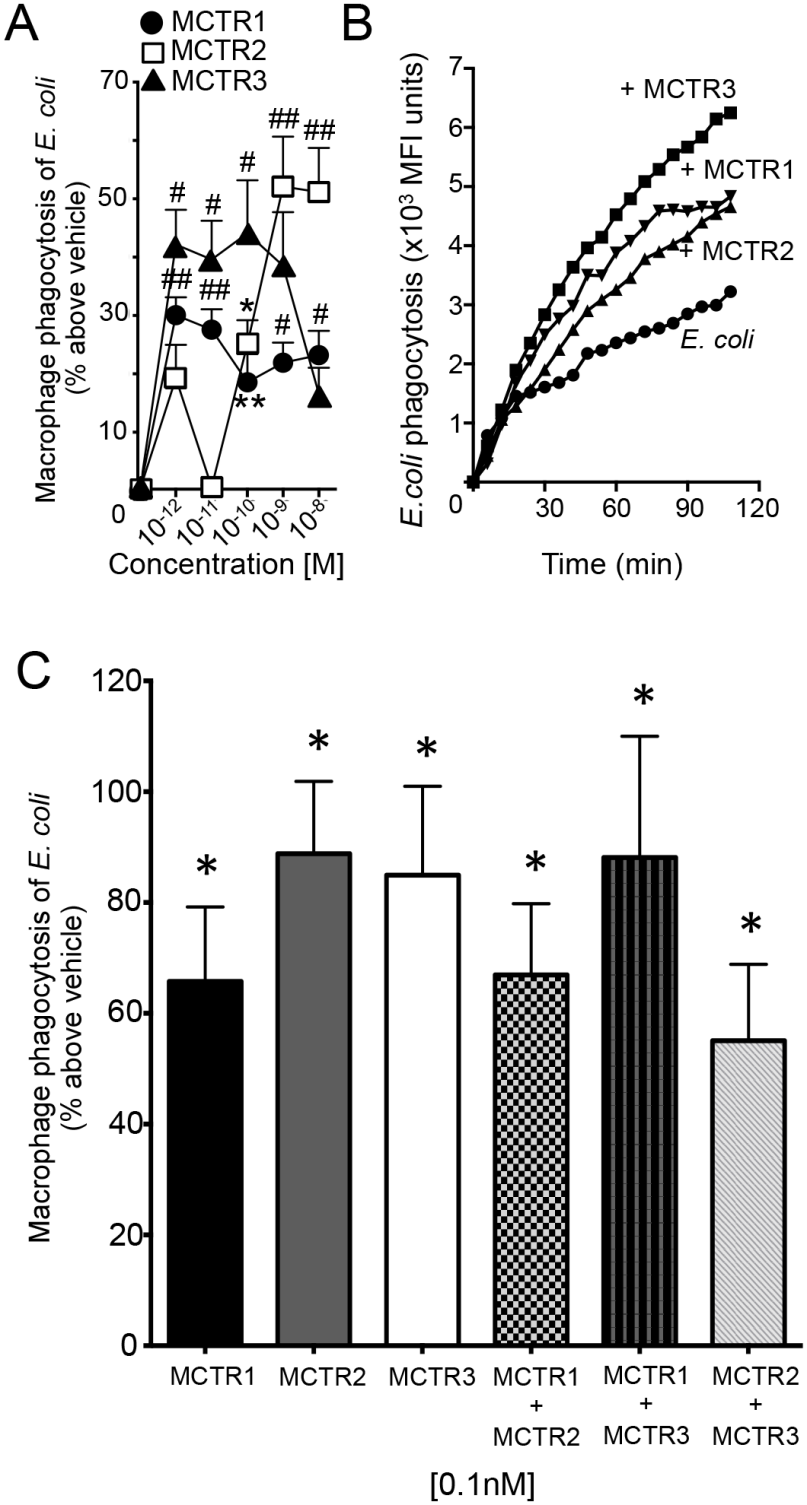
MCTRs are anti-inflammatory and pro-resolving with human phagocytes. Human macrophages (5 × 10^4^ cells per well) were incubated with vehicle (DPBS^+/+^), MCTR 1, MCTR2 or MCTR3 (at the indicated concentrations, 15 min, 37°C). Fluorescently labeled (A) *E*. *coli* (n = 8) were then added and phagocytosis assessed after 60min using a fluorescent plate reader. Results are mean ± SEM. *p<0.05, **p<0.01, #p<0.001, ##p<0.0001 vs. vehicle. (B) Human macrophages were plated onto chamber slides (6 x10^4^ cells/well) and incubated with MCTR1, MCTR2, MCTR3 (1 nM) or vehicle control (PBS^+/+^) for 15 min at 37°C, followed by addition of BacLight Green-labeled *E*. *coli*. Fluorescent images were recorded every 6 min for 120 min. In each experiment, 4 fields (20X) per condition (per well) were recorded. Results are mean fluorescence of 4 fields/well. n = 3 separate macrophage preparations. (C) *E*. *coli* phagocytosis by human macrophages incubated with MCTRs individually or the indicated combinations of MCTRs added simultaneously. Results are mean ± SEM for cells from n = 4 healthy human donors, *p<0.05 vs. vehicle (macrophages with *E*. *coli* alone).

Toward human translation, we validated the bioactions of these novel molecules with human primary leukocytes. Incubation of MCTR1 (1pM-10nM) with human macrophages gave dose-dependent increases (15–50%) in macrophage phagocytosis of *E*. *coli* with a maximum at ~1pM (Fig 4A). In addition, MCTR1 stimulated the efferocytosis of apoptotic neutrophils by macrophages (10–20% increase above vehicle; n = 8 macrophage preparations p<0.05), a key macrophage response in resolution cellular mechanisms [3, 4]. MCTR1, at a similar concentration range, also dose-dependently promoted phagocytosis of bacteria by human neutrophils with maxima at 100pM (30–70% increase above vehicle; p<0.05). Similar results were obtained for both MCTR2 (25–75%; p<0.05) and the new MCTR3 (35–50%; p<0.05; n = 4 neutrophil preparations).

Using live-imaging microscopy, we next investigated the kinetics of *E*. *coli* phagocytosis by human macrophages in order to gain insights into cellular mechanisms activated by MCTR. At 1nM, MCTR3 increased macrophage phagocytosis of fluorescently labeled *E*. *coli* as early as 30 min after addition; similar results were also obtained with MCTR1 and MCTR2 (Fig 4B). Of note, at the 2h interval the new MCTR3 gave the highest increase in *E*. *coli* phagocytosis followed by MCTR1 and MCTR2. These results demonstrate that MCTRs display potent anti-inflammatory and pro-resolving actions where MCTR3 gave highest activity with human macrophage phagocytic responses (MCTR3>MCTR1>MCTR2). While each of the MCTRs proved to be a statistically significant and potent activator of macrophage phagocytosis (e.g. efferocytosis) on its own, when added together none of the MCTR displayed synergy (Fig 4c) in this critical resolution response.

MCTRs also regulated human neutrophil responses. Each of these molecules dose-dependently regulated human neutrophil phagocytosis of *E*. *coli* with MCTR3 displaying higher potency at promoting neutrophil phagocytosis at doses as low as 1pM-100pM, with ~60% increase in phagocytosis when compared to both MCTR2 and MCTR3 (S1 Fig). MCTR1 and MCTR3 each gave a bell-shaped curve that is characteristic of G protein-coupled receptor activation by SPM [17] that may reflect cellular shape changes at higher concentrations of the mediators. MCTR2 gave two distinct peaks, suggesting this mediator may activate two distinct receptors, a high affinity and low affinity receptor, on macrophages that mediate its actions in regulating phagocytosis of *E*. *coli*.

## Discussion

The present findings establish the complete stereochemistry of the first MCTR: MCTR1 as 13R-glutathionyl, 14S-hydroxy-4Z,7Z,9E,11E,13R,14S,16Z,19Z-docosahexaenoic acid, for MCTR2 as 13R-cysteinylglycinyl, 14S-hydroxy-4Z,7Z,9E,11E,13R,14S,16Z,19Z-docosahexaenoic acid and that for the new MCTR3 as 13R-cysteinyl, 14S-hydroxy-4Z,7Z,9E,11E,13R,14S,16Z,19Z-docosahexaenoic acid, and their bioactions. These results also confirm the potent biological actions of MCTR1 and MCTR2 originally uncovered [2] and now demonstrate that the new member of the series MCTR3 carries potent, unanticipated bioactions governing the cardinal signs of resolution, namely clearance of debris and infections by phagocytes, tissue regeneration, and regulation of pro-inflammatory chemical mediators [3]. Together, these results assess the overall rank order potencies of MCTRs at promoting the resolution of live bacterial infections as MCTR3 ≥ MCTR2 > MCTR1 (see Fig 4).

Highly effective bioactive metabolic structure-function units are retained and copied throughout evolution. This is exemplified by the diverse roles of the isoprenoid unit throughout the biosphere with many biosynthetic pathways and wide ranging functions. Indeed this structural unit is the building block of many biologically significant molecules including steroids, retinoids and terpenoids [18]. By comparison, the enzymatically-produced allylic epoxides (Fig 1) central to maresin and SPM biosynthesis also appear to be highly conserved in chemical evolution as a central building block in the biosynthesis of diverse bioactive molecules with wide ranging functions such the MCTR from 13S,14S-eMAR [2] and, for example, CysLT from the epoxide intermediate leukotriene A_4_ [5, 19]. Of relevance to our present studies is the conjugation of allylic epoxides with sulfur-containing peptides that give rise to molecules that carry potent infection-resolution properties (shown in Fig 1). The biological actions of these molecules are also separate, whereby the cysteinyl leukotrienes carry potent broncho-constrictive and inflammation-initiating actions [5, 20], whereas MCTR are potently pro-resolving and function in tissue regeneration [2, 3]. MCTR3 enhanced both human PMN and macrophage responses (Fig 4 and S1 Fig) yet did not appear to display synergy or alter PMN survival *in vitro* in these conditions (S1 Table). Of interest, MCTR3 proved to be a potent bioactive molecule with planaria, mouse, and human cells. Thus the present findings provide new, highly conserved targets and pathways linking resolution of infections with tissue regeneration.

## Supporting Information

S1 Fig MCTRs enhance human PMN phagocytosis and human macrophage efferocytosis of apoptotic PMN. (A) Human neutrophil phagocytosis. Human neutrophils (1 × 10^5^ cells per well) were incubated with vehicle (DPBS^+/+^), MCTR 1, 2 or 3 (at the indicated concentrations, 15 min, 37°C). Fluorescently labeled *E*. *coli* (1:50) was then added and phagocytosis assessed after 1 hour (n = 4). Results are mean ± SEM. *p<0.05 vs. vehicle; determined using One-way ANOVA. (B) Human macrophage efferocytosis. Human macrophages (5 × 10^4^ cells per well) were incubated with vehicle (DPBS^+/+^), MCTR 1, MCTR2 or MCTR3 (at the indicated concentrations, 15 min, 37°C). Fluorescently labeled apoptotic PMN (n = 5) were then added and phagocytosis assessed after 60min using a fluorescent plate reader. Results are mean ± SEM. *p<0.05, **p<0.01, #p<0.001, ##p<0.0001 vs. vehicle.
(PDF)

S1 Table MCTRs and human PMN Survival in vitro. Human PMN were incubated for either 24 or 48 hours in DPBS^+/+^ (pH 7.2, 37°C, 5% CO_2_) in vehicle (DPBS^+/+^ alone) or with the addition of 10nM MCTR1, MCTR2, or MCTR3 at time zero. Cell viability was assessed using flow cytometry with surface staining for Annexin V and Propidium Iodide. Results are mean ± SEM from n = 3 healthy volunteers. Annexin^+^, PI^−^represents early apoptotic PMN and Annexin^+^, PI^+^ represents late apoptotic PMN populations. There were no statistically significant differences in apoptotic populations between vehicle (DPBS^+/+^ alone) and MCTR treated PMN by one-tailed T-test.
(PDF)

## Abbreviations

LM: lipid mediator
MCTR: maresin conjugates in tissue regeneration
MCTR1: 13-glutathionyl, 14-hydroxy-docosahexaenoic acid
MCTR2: 13-cysteinylglycinyl, 14-hydroxy-docosahexaenoic acid
MCTR3: as 13-cysteinyl, 14-hydroxy-docosahexaenoic acid
R_*i*_: resolution interval
SPM: specialized pro-resolving mediators
T_50_: time to 50% regeneration
T_R_: retention time
TRI_max_: maximum tissue regeneration
