# Supplementary figures and images for: Identification and Actions of a Novel Third Maresin Conjugate in Tissue Regeneration: MCTR3
Source: PLoS One. 2016 Feb 16;11(2):e0149319. doi: 10.1371/journal.pone.0149319 (PMC4755597; doi:10.1371/journal.pone.0149319)

**A**

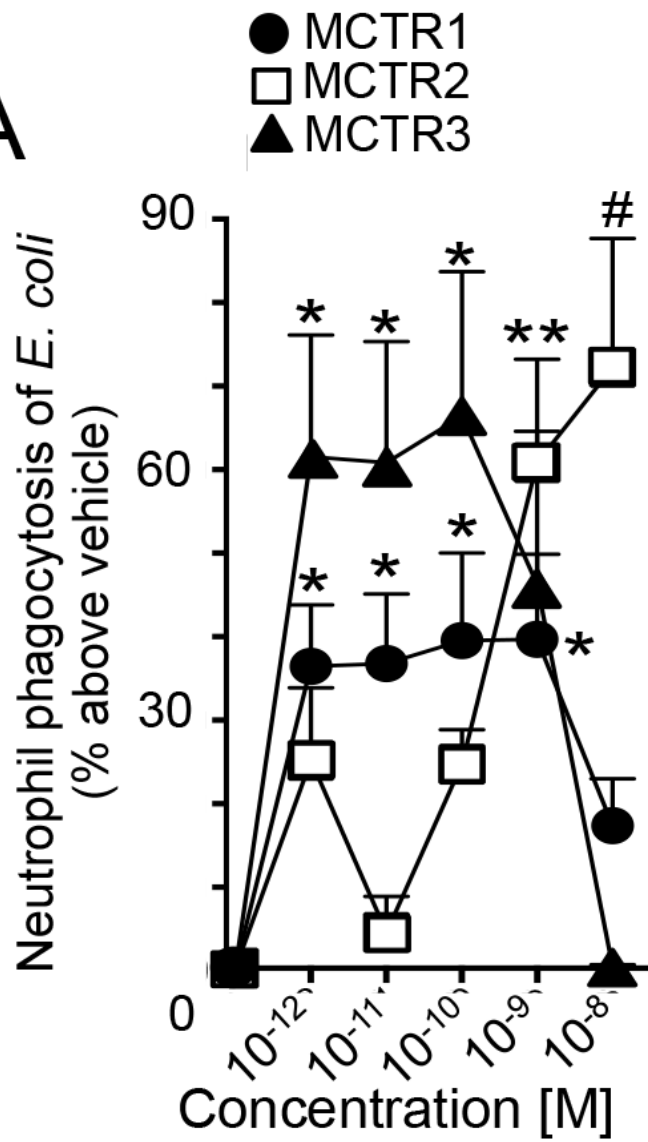

**B**

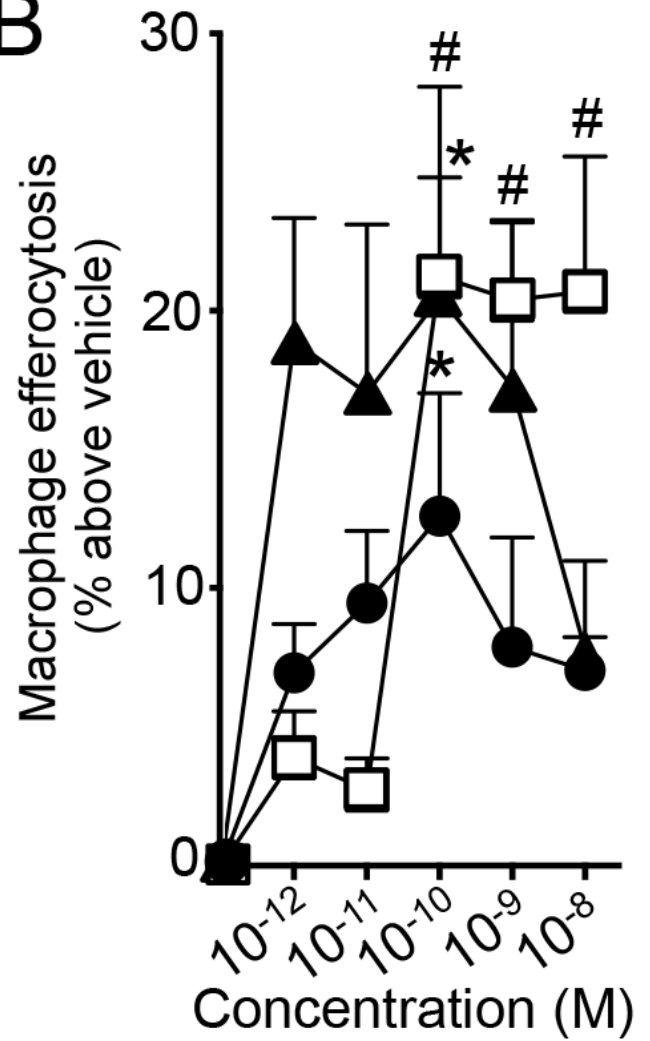

Supplement: S1 Fig — (A) Human neutrophil phagocytosis. Human neutrophils (1 × 10^5^ cells per well) were incubated with vehicle (DPBS+/+), MCTR 1, 2 or 3 (at the indicated concentrations, 15 min, 37°C). Fluorescently labeled E. coli (1:50) was then added and phagocytosis assessed after 1 hour (n = 4). Results are mean ± SEM. *p<0.05 vs. vehicle; determined using One-way ANOVA. (B) Human macrophage efferocytosis. Human macrophages (5 × 10^4^ cells per well) were incubated with vehicle (DPBS+/+), MCTR 1, MCTR2 or MCTR3 (at the indicated concentrations, 15 min, 37°C). Fluorescently labeled apoptotic PMN (n = 5) were then added and phagocytosis assessed after 60min using a fluorescent plate reader. Results are mean ± SEM. *p<0.05, **p<0.01, #p<0.001, ##p<0.0001 vs. vehicle. (PDF) [file pone.0149319.s001.pdf]
