# Supplementary material for: Identification and Actions of a Novel Third Maresin Conjugate in Tissue Regeneration: MCTR3
Source: PLoS One. 2016 Feb 16;11(2):e0149319. doi: 10.1371/journal.pone.0149319 (PMC4755597; doi:10.1371/journal.pone.0149319)
Supplement: S1 Table — Human PMN were incubated for either 24 or 48 hours in DPBS+/+ (pH 7.2, 37°C, 5% CO2) in vehicle (DPBS+/+ alone) or with the addition of 10nM MCTR1, MCTR2, or MCTR3 at time zero. Cell viability was assessed using flow cytometry with surface staining for Annexin V and Propidium Iodide. Results are mean ± SEM from n = 3 healthy volunteers. Annexin+, PI−represents early apoptotic PMN and Annexin+, PI+ represents late apoptotic PMN populations. There were no statistically significant differences in apoptotic populations between vehicle (DPBS+/+ alone) and MCTR treated PMN by one-tailed T-test. (PDF) [file pone.0149319.s002.pdf]

S1 Table. MCTRs and human PMN Survival in vitro [δ]

Time	Conditions	DPBS$^{+/+}$ alone	MCTR1	MCTR2	MCTR3
24 hours					
	Annexin^{+}, PI^{-} (%)	39.2 ± 8.3	39.9 ± 5.3	36.4 ± 8.6	37.8 ± 8.5
	Annexin^{+}, PI^{+} (%)	42.1 ± 10.9	44.9 ± 8.9	48.1 ± 12.3	47.6 ± 14.5
48 hours					
	Annexin^{+}, PI^{-} (%)	10.9 ± 2.8	6.9 ± 1.2	11.3 ± 4.4	12.8± 4.2
	Annexin^{+}, PI^{+} (%)	64.3 ± 15.2	70.0 ± 13.9	69.2 ± 17.8	67.5 ± 11.1

δ Human PMN were incubated for either 24 or 48 hours in DPBS$^{+/+}$ (pH 7.2, 37°C, 5% CO_2) in vehicle (DPBS$^{+/+}$ alone) or with the addition of 10nM MCTR1, MCTR2, or MCTR3 at time zero. Cell viability was assessed using flow cytometry with surface staining for Annexin V and Propidium Iodide. Results are mean ± SEM from n=3 healthy volunteers. Annexin^{+}, PI^{-} represents early apoptotic PMN and Annexin^{+}, PI^{+} represents late apoptotic PMN populations. There were no statistically significant differences in apoptotic populations between vehicle (DPBS$^{+/+}$ alone) and MCTR treated PMN by one-tailed T-test.
